# Clinician knowledge and attitudes of mental health advance statements in Victoria, Australia

**DOI:** 10.1111/inm.13022

**Published:** 2022-05-20

**Authors:** Russell James, Phil Maude, Adam Searby

**Affiliations:** ^1^ School of Nursing, College of Health and Medicine University of Tasmania Hobart Tasmania Australia; ^2^ La Trobe Rural Health School, Violet Vines Marshman Centre for Rural Health Research Latrobe University Melbourne Victoria Australia; ^3^ School of Nursing & Midwifery Deakin University, Institute for Health Transformation Geelong Victoria Australia

**Keywords:** advance directives, advance statements, health services, mental health, psychiatric wills

## Abstract

Embedded into Victoria's mental health legislation as part of the 2014 Mental Health Act suite of reforms, advance statements are designed to convey an individuals' preferences for treatment during times when the ability to communicate or make decisions may be impaired. This study investigated Victorian mental health clinicians' knowledge and attitudes of advance statements as well as their experience with training and implementation. We used an online Qualtrics survey of Victorian mental health clinicians (*n* = 190) to achieve this aim. Instrument validity was determined using the Content Validity Index (CVI) with field experts rating each item for relevance. A value of 80% or higher was sought and computed for each individual item on the scale, as well as for the overall scale. The Cronbach's Alpha coefficient was conducted to determine internal consistency reliability with a value of α = 0.721 for the survey, suggesting that the scale had acceptable internal consistency and reliability. Despite widespread support and positive attitudes towards advance statements existing among mental health clinician participants, the level of knowledge and perception of barriers continues to significantly affect the wide‐spread uptake of advance statements. The quality and extent of training in legal and clinical aspects of advance statement varied widely among the study participants, with the quality and benefits of the training affecting participant reported confidence level as well as their practical experience with advance statements. Three recommendations can be made: that advance statements are embed into routine mental health practice to identify individuals who have existing advance statements and support those who do not to prepare one; that regular co‐produced and facilitated training be provided to increase understanding, promotion, and overall use and uptake of advance statements; and finally, for local mental health service to develop a culture for positive engagement and promotion of autonomy through inclusive practices around decision‐making.

## INTRODUCTION

Advance statements, also known internationally as “psychiatric advance directives” and “psychiatric wills”, are a means of promoting and facilitating choice, empowerment, and autonomy, and have been implemented in many global jurisdictions (Weller 2010). Under mental health legislation in Victoria, Australia (the setting for this study), advance statements are defined as “… a document that sets out a person's preferences in relation to treatment in the event that the person becomes a patient” (Mental Health Act 2014 p.26). Advance statements were introduced into Victoria as part of a suite of reforms underpinning the introduction of the 2014 Mental Health Act. The overarching aim of advance statements is to enable collaboration between individuals accessing mental health services and the services themselves by assisting individuals to participate in decisions about their treatment (Bogdanoski 2009; Borschmann *et al*. 2014; Farrelly *et al*. 2015). Advance statements achieve this aim by providing a mechanism for people to express their preferences of care, and how they wish to be treated, compared to a directive which has legally binding requirements (Miller 2017). In this paper, we aim to identify mental health clinician knowledge and attitudes towards the use of advance statements and their experience with training and implementation in the state of Victoria, Australia.

## BACKGROUND

Implemented in July 2014, the Victorian *Mental Health Act* introduced many significant changes to the methods of compulsory assessment and treatment of people living with mental illness (Maylea *et al*. 2021). The *Act* actively encourages mental health service users to be fully involved in their own mental health support, treatment, and self‐management (s 10(d)). Further, one of the primary goals of the *Act* is to ensure that people living with mental illness, who are subject to involuntary treatment, are supported to make and participate in decisions about their mental health treatment (s 11(1)(c)). The inclusion of the advance statement (ss 19–22) and nominated person (ss 23–26) model (i.e. proxy decision‐maker) within the legislation was a first for Australia and was a paradigm shift from State‐directed substitute decision‐making when a compulsory treatment order has been applied to an individual (Turton‐Lane & Clarke 2014). The inclusion of advance statements within mental health legislation is acknowledged as a practice change that recognizes how mental health providers engage individuals who encounter mental health services, and value their preferences to care received (James *et al*. 2019). However, the familiarity and knowledge mental health clinicians have of advance statements greatly affects their implementation within the mental health setting (James *et al*. 2022).

Several studies investigating mental health advance planning tools identified barriers of use and implementation (Shields *et al*. 2014), including a lack of ability to access documents (Kim *et al*. 2007, 2008; Srebnik & Brodoff 2003; Van Dorn *et al*. 2006), inadequate knowledge, and awareness of the existence of advance statements (Elbogen *et al*. 2006; O'Connell & Stein 2005), poor training opportunities (Wilder *et al*. 2013), issues with communication (Henderson *et al*. 2010), time constraints (Van Dorn *et al*. 2006), or willingness to share decision‐making responsibilities with services uses (Atkinson *et al*. 2004; Kim *et al*. 2008), and clinician attitude towards advance planning tools (Henderson *et al*. 2010).

### Attitudes towards the use of advance statements

The attitudes of mental health clinicians towards the use and implementation of advance statements are crucial to their effective use, as it is mental health clinicians who have the potential to be involved in each stage of the advance statement (i.e. preparation, creation, and implementation during a mental health crisis) (Van Dorn *et al*. 2006). Widespread support for planning tools remains within the mental health setting and the adoption of inclusivity and inclusion of proxy decision‐makers in decisions for mental health care and treatment (Van Dorn *et al*. 2006). However, substantial barriers remain towards the practical application of advance planning tools within the clinical setting (Wilder *et al*. 2013). Frontline worker resistance and reluctance to share power with recipients of care and health care providers' own attitudes and values they bring to their own practice have a marked influence on decisions (Kim *et al*. 2008). In addition, mental health providers decision making is influenced by competing values when working with mental health service users. Often facing ethical challenges, like deciding to admit an individual under mental health legislation or balancing an individual's right to self‐determination and autonomy against concerns for the person's mental wellbeing, and service constraints (Kim *et al*. 2008).

### Knowledge of advance statements

The familiarity and knowledge mental health clinicians have of advance statements greatly affects their implementation within the mental health setting (Shields *et al*. 2014). Several studies have identified factors to the implementation of advance planning tools within the mental health setting (Shields *et al*. 2014; Van Dorn *et al*. 2006; Wilder *et al*. 2013). However, only a small number of studies have explored mental health workers knowledge and understanding of advance planning tools within the mental health setting. Differences in definition and legislation across jurisdictions results in difficulty in comparing circumstances. While health care professionals are increasingly aware of advance statements, the practical use of advance statements is not common in the mental health setting (Borbe *et al*. 2012; Radenbach *et al*. 2014). The notion that mental health clinicians have a theoretical familiarity but lacked hands‐on clinical experience of advance statements (Gieselmann *et al*. 2018) has led to a critical attitude towards advance statements and overall impact upon implementation efforts (Van Dorn *et al*. 2006). A lack of clinician knowledge of advance statements, and limited training opportunities for mental health clinicians, has a direct correlation between the barriers perceived by clinicians and their actual knowledge about advance planning tools (Wilder *et al*. 2013). The use of advance statement within the mental health setting creates challenges in educating and engaging people who encounter mental health service on their use. Poor knowledge among health care providers affects the speed and effectiveness of implementation, with training and education of health care providers being vital to the success of implementation. Embedding advance statements into routine care using ‘champions’, identified as interested and engaged members of staff invested in the implementation has resulted in some good outcomes (Durlak & DuPre 2008; Kemp *et al*. 2015). Here, staff would be involved in leadership of the co‐ordination process and serve to maintain the agency's focus on and commitment to the implementation of legislation into practice, within health services. Staff in these roles could best maintain momentum and facilitate the education of all mental health staff and service users.

## AIM

The aim of this study was to identify mental health clinician knowledge and attitudes towards the use of advance statements in the state of Victoria, Australia. This aim reflected one of the larger studies research questions which was: What knowledge and attitudes do mental health clinicians have of advance statements?

## DESIGN

An online survey was used to capture the opinions of clinicians working in Victoria, Australia, towards advance statements and their perceived knowledge and attitudes of their implementation and use. A demographic profile and estimated experience with advance statements were also obtained. Ethical approval for this study was granted by the relevant university ethics committee prior to data collection commencing. This study is reported in accordance with the Transparent Reporting of Evaluations with Nonrandomized Designs (TREND) statement checklist (Des Jarlias *et al*. 2004).

### Instrument design

The researchers investigated the use of survey instruments used in previous similar studies, concluding that these tools did not adequately address the research focus of this study, and could not be adapted to the Victorian mental health landscape. This was due to the nature of the advance statement model within Victoria, and the study focus to best identify and understand the knowledge and attitudes of Victorian mental health clinicians. Therefore, it was necessary to develop a new survey tool, based on existing instruments identified from the literature (Thom *et al*. 2015; Wilder *et al*. 2013), that specifically addressed the research questions.

The instrument developed for use in a larger mixed methods study consisted of 50 items, with a demographic section, followed by question responses using an ordinal, four‐point Likert type scale. This paper reports the results of item questions that aimed to explore participant knowledge and attitudes of advance statement implementation and use. A four‐point scale was used as the research aimed to capture participants' attitudes, and opinions, thus a middle taking of “no opinion” was not provided as an option (Lynn 1986; Polit & Beck 2006). Table 1 presents the survey questions of the relevant items.

**Table 1:** Survey questions of the relevant items

Since the implementation of the 2014 Victorian Mental health Act, have you received training on advance statements?	
How long was the training you attended	Less than 1 h
	1–2 Hours
	2–4 Hours
	5–8 Hours
	Greater than 8 Hours
Do you believe that advance statements are needed within the mental health setting?	
Please indicate below your opinion towards the following statements regarding knowledge of, and training in advance statements	
I have received training in advance statements	
Training I attended exceeded my expectations	
I have adequate knowledge to initiate discussions for the use of an advance statement with individuals	
I feel confident to execute an advance statement	
Please indicate below your opinion towards the following statements regarding attitudes around Advance Statements	
Advance statements have the potential to influence treatment decisions	
Clinicians assisting individuals to complete an advance statement have the potential to influences their treatment preferences	
It is not my role to assist individuals about the use of advance statements	
Mental health service users do not have adequate knowledge of advance statements	
Advance statements will be used by individuals to refuse all medications	
Advance statements will be used by individuals to refuse all treatments	
Advance statements are a waste of time	
Advance statements are often disregarded due to MH legislation being able to override preferences	
Advance statements have the potential to bring an unnecessary risk of violence and aggression to MH staff if treatment preferences are unable to be honoured	

### Validity

Content validity is defined as the extent to which an instrument adequately samples the research domain of interest when attempting to measure phenomena (Carmines & Zeller 1979; Waltz *et al*. 2005). A content validity panel was assembled with five members identified as experts in the field of mental health practice. Panel members were asked to read each item in the survey and evaluate the relevance of each item to the research question of the study using a separate item rating form (Grant & Davis 1997).

A widely used method of quantifying content validity for multiitem scales is the Content Validity Index (CVI), which is based on field experts rating each item for relevance (Polit *et al*. 2007). The CVI value was computed for each individual item on the scale, as well as for the overall scale. The scoring system for each item (the I‐CVI) is computed as the number of experts giving a rating of either 3 or 4, divided by the of number experts. Items were included if they scored 0.80 or greater, which was used to determine the lower limit for acceptability of inclusion in the instrument design. The 80% threshold, supported by Davis (1992), was adopted for this content validity exercise.

### Reliability of the tool

The internal consistency reliability of the overall questionnaire was determined using the Cronbach's Alpha coefficient. A Cronbach's Alpha coefficient was conducted to determine internal consistency reliability with a value of α = 0.721 for the survey, suggesting that the scale had acceptable internal consistency and reliability (Taber 2018).

### Data collection

A convenience sampling method was used to recruit participants for the online survey. Participants who were currently working in the Victorian mental health workforce were invited to participate in the research project using a targeted email and flyers placed in physical workplace locations. The flyer provided a link to a Qualtrics online survey which included the Participant Information Sheet. The inclusion criteria for this study required that individuals were currently working within Victorian mental health services as a health professional, or within a government department or consumer group connected with a mental health service. They needed to be over 18 years of age. Invitations to participate were distributed through the communication channels of the office of the Chief Mental Health Nurse at the Victorian Department of Health and Human Services. A snowball sampling approach was used by disseminating survey invitations through professional organizations, at conferences, and through the professional organization. The data collection period was between July 2017 and February 2018. Because this was a one‐off explorative online survey with all clinicians across Victoria eligible, sample size calculation was not conducted.

### Data analysis

Data screening was conducted before analysis and missing data excluded from statistical procedures. Data from the online surveys was entered to SPSS v26 (IBM Corporation 2019). Descriptive statistics were sought for each item, with internal consistency and reliability established. Responses were explored and visualized using descriptive statistics, with nominal and ordinal variables presented as counts and percentages and interval and or ratio variables presented as means and standard deviation. Spearman's Correlation was used to examine for any correlation between survey items as variables. Due to the difference between subgroup sizes and the relatively small sample size of each subgroup, we used non‐parametric testing to explore descriptive data (Corder & Foreman 2011).

## RESULTS

### Demographic data

A total of 190 mental health clinicians participated in this study. Most of the respondents were Mental Health Nurses (*n* = 125, 62%), followed by Allied Health Professionals (*n* = 31, 15%) (Occupational Therapy *n* = 11, 5.5%; Social Work *n* = 11, 5.5%; Psychology *n* = 7, 3.5%; Pharmacist *n* = 1, 0.5%; Art Therapist *n* = 1, 0.5%), and Consumer/Carer Consultants (*n* = 15, 7.5%). The discipline of medicine was also represented (*n* = 14, 7%) (Psychiatrists *n* = 8, 4%, Psychiatric Registrars *n* = 5, 2.5%, and a Medical Officer *n* = 1, 0.5%). A small number (*n* = 5, 2.5%) of respondents identified as other, with representation from five professional groups (admin/ward clerk *n* = 1, Community Development *n* = 1; Compliance Coordinator *n* = 1; Mental Health Legal Advisor *n* = 1; Project Officer *n* = 1).

### Length of service and education

Across participants, the average length of service was 12.5 years (SD 9.876). Within each of the disciplines, the average length of service was 14.5 years for Mental Health Nurses (SD 10.688); 10.7 years for Medicine (SD 8.498); 9.7 years for Allied Health (SD 6.503); and 4.5 years for the Consumer/Carer Workforce (SD 2.809). Most participants had a graduate diploma (*n* = 67, 36%), followed by a master's degree (*n* = 64, 34%).

### Clinical setting

Most participants (85%) came from the public mental health setting (inpatient *n* = 61, community *n* = 101), with private mental health (*n* = 3, 2%), forensic mental health services (*n* = 3, 2%), and primary mental health including school, university, and education sectors (*n* = 4, 2%) being less represented. Table 2 presents an overview of participant demographic characteristics.

**Table 2:** Demographic profile

Characteristics	Sample (%)	*M*	*SD*
Profession			
*n*	190 (100%)		
Mental Health Nurse	125 (66%)		
Allied Health Professional	31 (16%)		
Psychiatry/Medical	14 (7%)		
Consumer/Carer Consultant	15 (8%		
Other	5 (3%)		
Length of Service (years)			
*n*	190 (100%)		
Mental Health Nurse	125 (66%)	12.5	9.976
Allied Health Professional	31 (16%)	14.5	10.688
Psychiatry/Medical	14 (7%)	9.7	6.503
Consumer/Care Worker	15 (8%)	10.7	8.498
Other	5 (3%)	4.5	2.809
Highest qualification			
*n*	187 (100%)		
Diploma	17 (9%)		
Bachelor's degree	34 (18%)		
Graduate diploma	67 (36%)		
Master degree	64 (34%)		
Doctor of Philosophy (PhD)	5 (3%)		
Employment Location			
*n*	190 (100%)		
Public Inpatient MH Unit	61 (32%)		
Public Community MH Unit	101 (52%)		
Private MH Service	3 (2%)		
Other	25 (14%)		

### Experience with advance statements

Participants were asked about their experiences in using and implementing advance statements. Of the responses to this question (*n* = 187), the majority (*n* = 80, 40%), of participants had no previous experience with advance statements. 44 (22%) indicated that they had experience in the preparation stage of an advance statement, whilst 19 (10%) indicated experience with the implementation of an advance statement. Similarly, 44 (22%) reported experience of both preparation and implementation.

Across the four groups, medical (*n* = 4, 29%) and allied health (*n* = 13, 48%) professions had the higher levels of practical experience of the preparation of advance statements within their professions than did mental health nurses (*n* = 22, 18%). Table 3 presents a breakdown of experience of advance statements among participants.

**Table 3:** Experience with advance statements

Characteristics	Sample (%)			
Total population				
*n*	187 (100%)			
Experience preparing AS	44 (22%)			
Experience implementing AS	19 (10%)			
Experience both preparing and implementing	44 (22%)			
No experience at all	80 (40%)			

Profession	MHN	Allied Health	Medical	Consumer/Carer Worker
*n*	123 (100%)	27 (100%)	14 (100%)	10 (100%)
Experience preparing AS	22 (18%)	13 (48%)	4 (29%)	1 (10%)
Experience implementing AS	15 (12%)	0 (0%)	3 (21%)	0 (0%)
Experience both preparing and implementing	28 (23%)	6 (22%)	3 (21%)	4 (40%)
No experience at all	58 (47%)	8 (30%)	4 (29%)	5 (50%)

### Knowledge and training

**Fig. 1:**
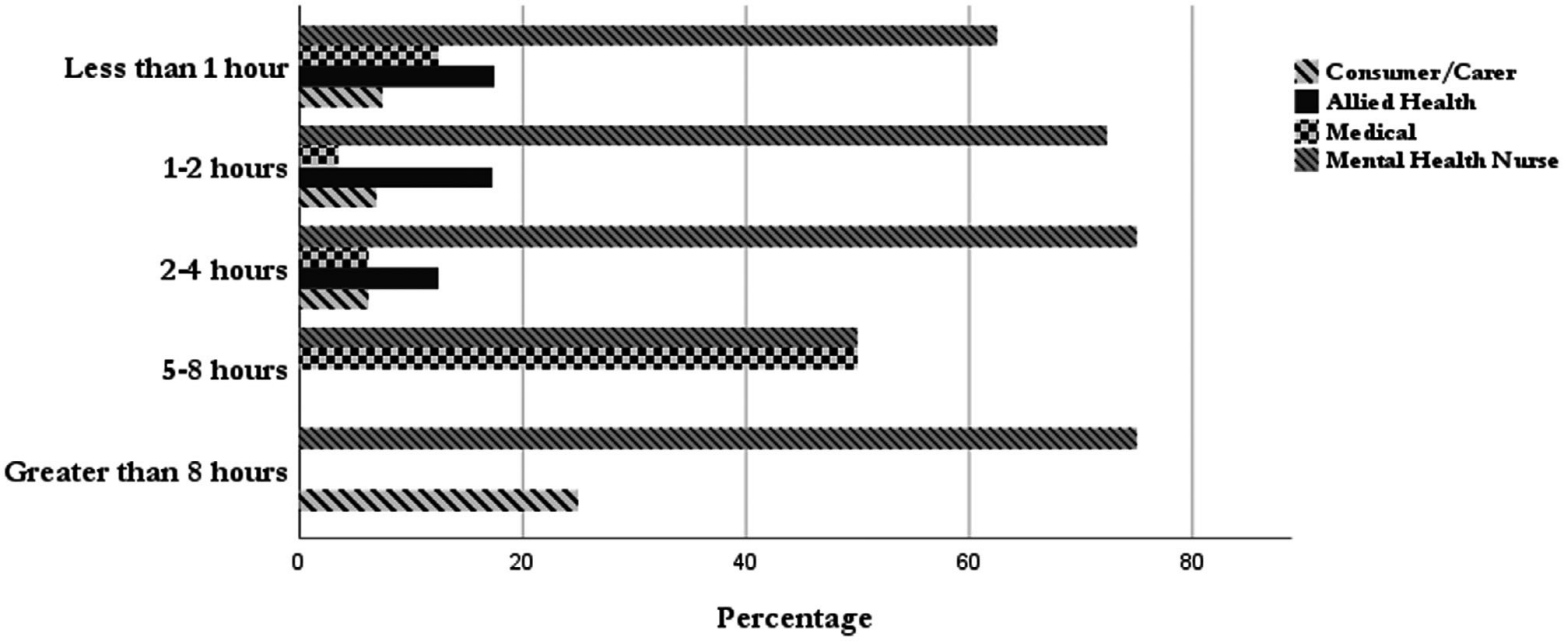
Duration of training attended.

Participants were asked to indicate if they had received advance statement training. Of the 190 respondents to the question, 63% (*n* = 127) reported that they had received training. Of the 127 respondents who had received training, the duration of the training ranged from <1 to >8 h. 49% (*n* = 102) of respondents received <2 h of training on advance statements. Figure 1 presents the breakdown of professional group duration of the training attended. Of note was the medical workforce who indicated they had received <1 h of training. A Spearman's Correlation was conducted to examine correlation between knowledge and confidence level of participants. The correlation was found to be significant (*rs* = 0.802, *P* = 0.001). A further correlation was conducted between training and the level of knowledge reported was also significant (*rs* = 0.44, *P* = 0.046).

Of the training received, 47% (*n* = 71) of respondents reported that training had met their needs and expectations, compared to 53% (*n* = 90) who reported that the training attended do not exceed their expectations.

**Fig. 2:**
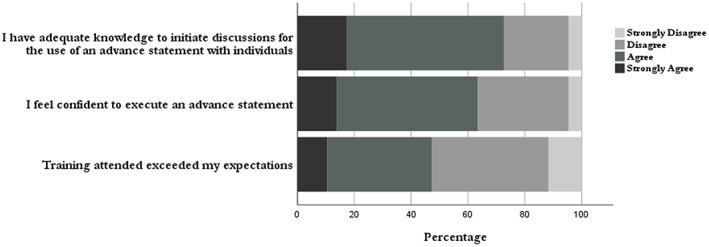
Perceptions of knowledge and training attended.

In relation to participant's knowledge of advance statements, two item questions were asked: “I have adequate knowledge to initiate discussions for the use of advance statements with individuals”; “I feel confident to execute an advance statement”. 73% (*n* = 123) of respondents reported that they felt they have adequate knowledge to initiate discussions for the use of advance statements, compared with 27% (*n* = 37) who did not. A slight fall in confidence was recorded in relation to individuals' level of confidence to execute the preferences of care detailed within an advance statement, with 63% (*n* = 109) of respondents indicating that they felt confident to execute an advance statement. Figure 2 presents the data from the Likert Scale responses.

### Attitudes

The positivity of participants towards the inclusion of advance statements within the mental health setting was universal with a majority (*n* = 175, 94%) believed that advance statements are needed within the mental health setting. Following this initial question, participants were asked nine subsequent item questions to rate (1 = Strongly Agree to 4 = Strongly Disagree) their opinion towards advance statements within the mental health setting (See Table 1 for each of the 9 item questions). Of the 171 participants who answered these questions, nearly all respondents (*n* = 158, 92%) believed that advance statements have the potential to influence treatment decisions. Furthermore, 77% (*n* = 132) of respondents agreed that clinicians who assist individuals to complete an advance statement have the potential to influence their treatment preferences. In terms of the roles and responsibilities of the mental health workforce, 88% (*n* = 151) disagreed with the statement that “it is not my role to assist individuals about the use of advance statements”, compared to 12% (*n* = 20) of respondents who agreed. Furthermore, when asked to indicate their level of agreement with the statement ‘Mental health service users do not have adequate knowledge of advance statements’, 88% (*n* = 151) of participants agreed.

Attitudes towards how advance statements could be used indicated that participants view an advance statement as a tool not to refuse treatments, rather to influence the decisions being made. Here, 76% (*n* = 148) of respondents disagreed with the item statement “advance statements will be used by individuals to refuse all medications” compared to 14% (*n* = 23) who agreed, and 77% (*n* = 149) with the statement that “advance statements will be used to refuse all treatments”, compared to 13% (*n* = 22) who felt that refusal of treatment was a key function.

The attitudes towards the use of advance statements within the mental health setting were also assessed, with a high majority of participants indicating that advance statements are not a waste of time and that in relation to the overriding capacity of mental health legislation 58% (*n* = 97) agreed that advance statements are often disregarded due to the mental health legislation being able to override an individual's preferences, compared to 42% (*n* = 71) who disagreed.

**Fig. 3:**
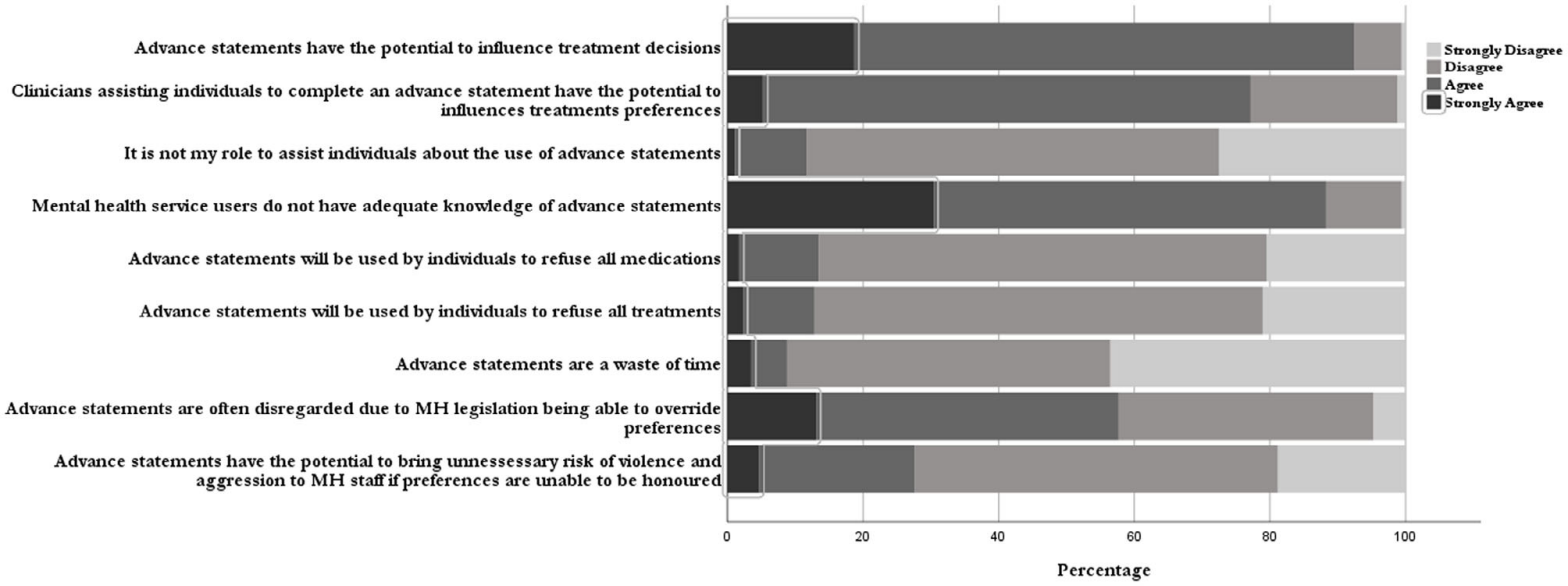
Attitudes towards advance statements.

In relation to attitudes towards the use of advance statements, 72% (*n* = 123) of the 170 respondents disagreed to the statement, “advance statements have the potential to bring an unnecessary risk of violence and aggression to mental health staff if treatment preferences are unable to be honoured”, compared to 28% (*n* = 47) who agreed. There was no correlation between professional groups, clinical setting and training attended relating to attitudes, with a consistent variation across participants towards the item responses relating to attitude finding no significance or standout measure. The results of all item responses in relation to participant attitude are presented in Figure 3.

## DISCUSSION

Our results indicate widespread support and positivity from mental health clinicians towards advance statements within the mental health setting. Many of the study's participants supported the implementation of advance statements as part of the Victorian legislation (Mental Health Act 2014); however, the level of knowledge of advance statements among mental health clinician participants remains a barrier to their use. The quality and availability of training remain crucial to how advance statements are used and supported (Henderson *et al*. 2010; Wilder *et al*. 2013). This study highlighted this, with participants reporting that training was limited in duration and content, and with half of those surveyed reporting that the training received did not meet expectations. The quality and extent of training about advance statements among mental health professionals are commonly found (Ambrosini *et al*. 2012; Amering *et al*. 1999; Elbogen *et al*. 2006; Kim *et al*. 2008; O'Connell & Stein 2005; Shields *et al*. 2014; Van Dorn *et al*. 2006; Wilder *et al*. 2013), with the views of participants that service users themselves do not have enough knowledge and understanding about advance statements is not uncommon.

The quality and extent of training in legal and clinical aspects of advance statement varied widely among the participants, with the quality and benefits of the training affecting the reported confidence level of participants, as did their practical experience with advance statements. A reluctance on the part of health professionals to discuss advance statements with service users is attributed to this (Srebnik & Brodoff 2003), as are increased concerns about potential barriers and negative attitudes towards the use of advance statements (Wilder *et al*. 2013). Without training across all levels of the mental health system by trained facilitators to assist individuals in completing advance statements, their implementation and use will continue to be impaired (Wilder *et al*. 2013; Swanson *et al*. 2006a, 2006b). The way training is delivered is also influential. It appears unlikely that the use of traditional workshop models or any single strategy will result in widespread success for the adoption of advance statements, so training delivery should comprise multiple overlapping techniques (Lyon *et al*. 2011). This combination of approaches must be selected to match the content of the intervention or practice being trained, the target audience, and the service setting (Beidas & Kendall 2010).

The majority of those surveyed had experience with advance statements, but 40% had not. Despite this finding, respondents indicated feeling confident to honour preferences detailed in an advance statement and said that overall, they felt they had adequate knowledge to initiate discussion with a service user about advance statements. Participants in this study reported that they had not had any experience with either the preparation or implementation of an advance statement, with almost half of those surveyed having had no experience of working with someone who had an advance statement. These findings indicate that advance statements have not been widely adopted into clinical practice, despite the potential advance statements have towards respecting individual autonomy and inclusion into decisions making (Henderson *et al*. 2008; Jankovic *et al*. 2010; Sellars *et al*. 2016; Swanson *et al*. 2006b; Weller 2010). Similar findings have been made elsewhere, with uptake of advance planning tools in the mental health setting remaining low (Amering *et al*. 2005; Shields *et al*. 2014). Previous research investigating the uptake of advance statements identifies that poor uptake is systemic due to the nature of the mental health setting, with substantial evidence that implementation of advance statements within the mental health setting is difficult and is affected by low rates of uptake and use (Swanson *et al*. 2006a), and low rates of access following completion (Henderson *et al*. 2010; Srebnik & Russo 2008).

The potential for advance statements to form part of routine practice is heartening, with support that advance statements have the potential to influence treatment decisions, providing a platform for conversations through a collaborative partnership to further understand individuals' personal beliefs, values, and wishes (Atkinson *et al*. 2004; Henderson *et al*. 2004; Morrissey 2015; Papageorgiou *et al*. 2002). In doing so, a shift in can occur in service delivery, creating an environment that embraces a culture of positivity and willingness to include individuals in their treatment decisions to produce better outcomes and achieve recovery and a more positive experience for all (Roviralto‐Vilella *et al*. 2019). This study's findings contribute to the recognition that using an advance statement enhances engagement and self‐management of illness, and enables individuals to take a greater responsibility for their care (Gergel & Owen 2015), in turn having a positive effect on health outcomes, with the view that advance statements are viewed as a tool not to create a problem in decision making for clinicians by refusing treatments, but rather to influence the decisions being made.

## RECOMMENDATIONS

The following recommendations, which may not necessarily be applicable across all areas within the mental health setting, arise from the study results and address several issues which could translate across mental health policy and health care delivery.

The primary recommendation is that advance statements must be established in routine mental health practice. For advance statements to be effective, and the benefits passed on to individuals, it is imperative that every person entering a mental health service be asked if they have an advance statement, if not, if they would like to create an advance statement, or, if so, whether their advance statement is current and accessible. This recommendation is supported by the findings, with participants indicating that use of advance statements is affected by time constraints and allocation of resources required to assist in a statement's completion.

Recommendations of training for advance statements stems from findings indicated that the training did not meet expectations and did not provide satisfactory information. Education and training at a local health care service level have the potential to improve the uptake and use of advance statements. Regular, co‐produced training with the active involvement of individuals with a lived experience of mental illness and treatment by mental health services would strengthen the training packages. Training should be tailored to identify and respond to the perspectives of individuals as to why a preference has been included, and to the sharing of experiences, which enables deeper understanding of advance statements in the clinical setting.

Another recommendation is aimed towards the local mental health service level, for it is at the grass roots where the approaches taken at a service level can greatly improve the uptake of advance statements. By increasing understanding of illness and treatments, increase autonomy and empowerment in the decision‐making processes, and improve service engagement and collaboration. Henderson *et al*.’s (2010) previous research supports this, arguing that all professionals within the mental health workforce must be equipped and prepared to engage and support individuals by informing, developing, and completing advance statements as well as to provide information about them. The clinical setting has the potential to drive advance statement use and inclusion through the adoption of favourable attitudes towards the advance statement model, accompanied by inquiry about advance statements, using positive and engaged practices, promoting the inclusion of advance statements into routine practice by explaining how they work, and ensuring that assistance is available to individuals. If these things are done, advance statement use will increase (Henderson *et al*. 2010).

## LIMITATIONS

Information to access the online survey was primarily distributed to mental health services and clinicians through Victoria's office of the Chief Mental Health Nurse and Chief Psychiatrist. Further, advertising and distribution was via professional organizations. This may have affected the demographic profile of those who completed the survey, resulting in the potential for response bias, as respondents self‐selected to partake in the survey and may have tended somewhat towards favourable views of advance statements. There was no ability to control for sample distribution or selection of participants in this anonymous online explorative survey.

The 190 returned surveys did facilitate satisfactory data analysis, but this sample size was not considered of a sufficient size to be representative of the Victorian mental health workforce. The sample was, however, diverse, as respondents came from several mental health settings, and their expertise was based on their experiences as mental health clinicians, consumer and carer consultants, educators, and advocates. Future studies should target a broader sample to ensure results can be generalized to a wider population.

As this survey tool was developed for use in an explorative study and gathered information relating to the participants' opinions towards advance statements in a cross‐sectional nature, future studies should use a longitudinal approach to measure changes in attitude over time.

## CONCLUSION

Advance statements have been used internationally to increase the autonomy and decision‐making authority of individuals accessing mental health services. Introduced as part of Victoria's mental health legislation as a way of supporting the rights of individuals receiving mental health treatment, advance statements are credited with improving mental health service user empowerment and self‐management, as well as improving communication between health care agencies and individuals, and collaboration within health care services. Victoria, being the first jurisdiction in Australia to adopt the advance statement model, has continued to be well placed nationally and internationally for the use of advance statements. This is due to the investment the Victorian government has made to enable and improve care and treatment for people living with mental illness.

As advance statements slowly make their way into the clinical landscape, the mental health system faces a formidable challenge. If change and further implementation are not enacted, the promise of advance statements effectiveness risks becoming diminished, rather than the good faith invitation to dialogue that mental health legislation and informants interpret it to be. Implementing new procedures is invariably felt to be cumbersome and artificial at the outset (Amering *et al*. 2005). Within this space, a great deal of trial and error is needed before workable, culturally congruent practices are arrived at. The findings of this study suggest that further deliberation, time, and well‐designed resources to support and inform decision‐making will be crucial to the successful implementation of advance statements in the mental health setting.

## RELEVANCE FOR CLINICAL PRACTICE

This study is the first Victorian study to survey the knowledge and attitudes of mental health clinicians towards the use of advance statements within the mental health setting. There is a need to understand the role and scope of how advance statements can be used within mental health services, how they best function, and to identify the barriers of service in the implementation of this initiative.

The current study adds to the body of knowledge on the challenges facing mental health services in adopting advance planning and empowering service users to play a greater role in treatment and care decisions and pathways.

## Data Availability

The data that support the findings of this study are available from the corresponding author upon reasonable request.
